# (*E*)-1-(2,2-Dimeth­oxy­eth­yl)-2-(nitro­methyl­idene)imidazolidine

**DOI:** 10.1107/S1600536810030266

**Published:** 2010-08-04

**Authors:** Dongmei Li, Zhongzhen Tian, Gaolei Wang, Peifeng Wei, Yanming Zhang

**Affiliations:** a Shandong Provincial Key Laboratory of Fluorine Chemistry and Chemical Materials, School of Chemistry and Chemical Engineering, University of Jinan, People’s Republic of China

## Abstract

In the title compound, C_8_H_15_N_3_O_4_, the 2-(nitro­methyl­ene)imidazolidine fragment is close to being planar (r.m.s. deviation = 0.027 Å), which may be correlated with delocalization of the electrons and the effect of the strongly electron-withdrawing NO_2_ group. An intra­molecular N—H⋯O link generates an *S*(6) ring. The same H atom also forms a weak inter­molecular N—H⋯O hydrogen bond, which results in *C*(7) chains propagating in [010].

## Related literature

For background to neonicotinoid insecticides, see Moriya *et al.* (1992 ▶). For the synthesis, see: Tian *et al.* (2007 ▶).
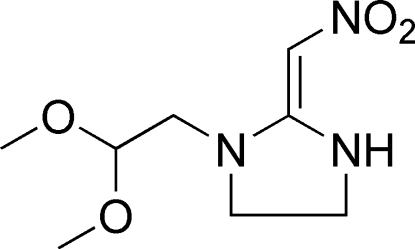


## Experimental

### 

#### Crystal data

C_8_H_15_N_3_O_4_
*M* *_r_* = 217.23
Monoclinic, 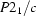

*a* = 10.444 (2) Å
*b* = 6.8676 (17) Å
*c* = 14.441 (3) Å
β = 99.953 (14)°
*V* = 1020.2 (4) Å^3^
*Z* = 4
Mo *K*α radiation
μ = 0.11 mm^−1^
*T* = 296 K
0.32 × 0.26 × 0.15 mm

#### Data collection

Bruker APEXII CCD diffractometer
Absorption correction: multi-scan (*SADABS*; Bruker, 2005 ▶) *T*_min_ = 0.965, *T*_max_ = 0.983
8629 measured reflections
2330 independent reflections
1849 reflections with *I* > 2σ(*I*)
*R*_int_ = 0.018

#### Refinement

*R*[*F* ^2^ > 2σ(*F* ^2^)] = 0.037
*wR*(*F* ^2^) = 0.110
*S* = 1.06
2330 reflections
139 parameters
H-atom parameters constrained
Δρ_max_ = 0.25 e Å^−3^
Δρ_min_ = −0.16 e Å^−3^

### 

Data collection: *APEX2* (Bruker, 2005 ▶); cell refinement: *SAINT* (Bruker, 2005 ▶); data reduction: *SAINT*; program(s) used to solve structure: *SIR97* (Altomare *et al.*, 1999 ▶); program(s) used to refine structure: *SHELXL97* (Sheldrick, 2008 ▶); molecular graphics: *SHELXTL* (Sheldrick, 2008 ▶); software used to prepare material for publication: *WinGX* (Farrugia, 1999 ▶).

## Supplementary Material

Crystal structure: contains datablocks I, global. DOI: 10.1107/S1600536810030266/hb5567sup1.cif

Structure factors: contains datablocks I. DOI: 10.1107/S1600536810030266/hb5567Isup2.hkl

Additional supplementary materials:  crystallographic information; 3D view; checkCIF report

## Figures and Tables

**Table 1:** Hydrogen-bond geometry (Å, °)

*D*—H⋯*A*	*D*—H	H⋯*A*	*D*⋯*A*	*D*—H⋯*A*
N2—H2⋯O2	0.86	2.09	2.6394 (17)	121
N2—H2⋯O3^i^	0.86	2.64	3.3554 (16)	141

Symmetry code: (i) 
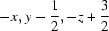
.

**Fig. 1.**
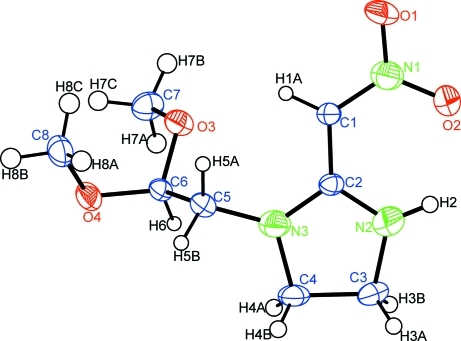
The molecular structure of (I) with displacement ellipsoids drawn at the 50% probability level. The H atoms are shown as circles of arbitrary size.

**Fig. 2.**
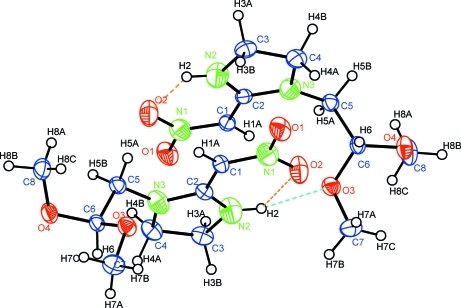
Intermolecular hydrogen bonding in the crystal structure of (I).

## supplementary crystallographic information

### Comment

Since the debut of Imidacloprid in 1990s (Moriya *et al.*, 1992), neonicotinoid insecticides have become rapidly an important chemical class of insecticides. Our interest was introducing oxygen atom into the lead struture and synthesizing a series of new compounds, in which the title compound (I) exhibited good insecticidal activities against pea aphids.

The structure of (I) is shown in Fig. 1 with the atom-numbering scheme. The delocalization of the electrons as far as the strong electron-withdrawing group, NO_2_, lead to a coplanar olefin-amine π-electron network. Intermolecular hydrogen bonds (N2—H2···O3) are found, and link the molecules into chains.

### Experimental

The title compound was synthesized according to the literature (Tian *et al.*, 2007).  Colourless prisms of (I) were obtained by slow evaporation of the solution of dichloromethane and ethyl acetate of the title compound.

### Refinement

H atoms bonded to N and O atoms were located in a difference map and refined with distance restraints of N—H = 0.87 (2) Å, and with *U*_iso_(H) = 1.2*U*_eq_(N). Other H atoms were positioned geometrically and refined using a riding model (including free rotation about the ethanol C—C bond), with C—H = 0.95–0.99 Å and with *U*_iso_(H) = 1.2 (1.5 for methyl groups) times *U*_eq_(C).

### Crystal data

**Table:**

C_8_H_15_N_3_O_4_	*F*(000) = 464
*M**_r_* = 217.23	*D*_x_ = 1.414 Mg m^−^^3^
Monoclinic, *P*2_1_/*c*	Mo *K*α radiation, λ = 0.71073 Å
Hall symbol: -P 2ybc	Cell parameters from 3493 reflections
*a* = 10.444 (2) Å	θ = 2.9–27.3°
*b* = 6.8676 (17) Å	µ = 0.11 mm^−^^1^
*c* = 14.441 (3) Å	*T* = 296 K
β = 99.953 (14)°	Prism, colourless
*V* = 1020.2 (4) Å^3^	0.32 × 0.26 × 0.15 mm
*Z* = 4	

### Data collection

**Table:**

Bruker APEXII CCD diffractometer	2330 independent reflections
Radiation source: fine-focus sealed tube	1849 reflections with *I* > 2σ(*I*)
graphite	*R*_int_ = 0.018
φ and ω scans	θ_max_ = 27.6°, θ_min_ = 2.0°
Absorption correction: multi-scan (*SADABS*; Bruker, 2005)	*h* = −13→13
*T*_min_ = 0.965, *T*_max_ = 0.983	*k* = −8→8
8629 measured reflections	*l* = −18→18

### Refinement

**Table:**

Refinement on *F*^2^	Secondary atom site location: difference Fourier map
Least-squares matrix: full	Hydrogen site location: inferred from neighbouring sites
*R*[*F*^2^ > 2σ(*F*^2^)] = 0.037	H-atom parameters constrained
*wR*(*F*^2^) = 0.110	*w* = 1/[σ^2^(*F*_o_^2^) + (0.0508*P*)^2^ + 0.1844*P*] where *P* = (*F*_o_^2^ + 2*F*_c_^2^)/3
*S* = 1.06	(Δ/σ)_max_ < 0.001
2330 reflections	Δρ_max_ = 0.25 e Å^−^^3^
139 parameters	Δρ_min_ = −0.16 e Å^−^^3^
0 restraints	Extinction correction: *SHELXL97* (Sheldrick, 2008), Fc^*^=kFc[1+0.001xFc^2^λ^3^/sin(2θ)]^-1/4^
Primary atom site location: structure-invariant direct methods	Extinction coefficient: 0.014 (2)

### Special details

**Table:**

Geometry. All e.s.d.'s (except the e.s.d. in the dihedral angle between two l.s. planes) are estimated using the full covariance matrix. The cell e.s.d.'s are taken into account individually in the estimation of e.s.d.'s in distances, angles and torsion angles; correlations between e.s.d.'s in cell parameters are only used when they are defined by crystal symmetry. An approximate (isotropic) treatment of cell e.s.d.'s is used for estimating e.s.d.'s involving l.s. planes.
Refinement. Refinement of *F*^2^ against ALL reflections. The weighted *R*-factor *wR* and goodness of fit *S* are based on *F*^2^, conventional *R*-factors *R* are based on *F*, with *F* set to zero for negative *F*^2^. The threshold expression of *F*^2^ > σ(*F*^2^) is used only for calculating *R*-factors(gt) *etc*. and is not relevant to the choice of reflections for refinement. *R*-factors based on *F*^2^ are statistically about twice as large as those based on *F*, and *R*- factors based on ALL data will be even larger.

### Fractional atomic coordinates and isotropic or equivalent isotropic displacement parameters (Å^2^)

**Table:**

	*x*	*y*	*z*	*U*_iso_*/*U*_eq_	
C1	0.00359 (12)	0.08330 (19)	0.63950 (8)	0.0427 (3)	
H1A	0.0562	0.0860	0.5937	0.051*	
C2	0.06233 (12)	0.08637 (17)	0.73529 (8)	0.0406 (3)	
C3	0.09094 (16)	0.0794 (2)	0.89827 (9)	0.0563 (4)	
H3A	0.0816	−0.0387	0.9333	0.068*	
H3B	0.0774	0.1913	0.9364	0.068*	
C4	0.22254 (15)	0.0880 (2)	0.86762 (9)	0.0597 (4)	
H4A	0.2701	0.2036	0.8921	0.072*	
H4B	0.2741	−0.0262	0.8888	0.072*	
C5	0.29097 (12)	0.08628 (19)	0.70666 (9)	0.0461 (3)	
H5A	0.2533	0.0386	0.6447	0.055*	
H5B	0.3573	−0.0057	0.7342	0.055*	
C6	0.35394 (11)	0.28135 (19)	0.69663 (8)	0.0414 (3)	
H6	0.3643	0.3483	0.7574	0.050*	
C7	0.31179 (18)	0.5845 (2)	0.62367 (12)	0.0641 (4)	
H7A	0.3323	0.6410	0.6853	0.096*	
H7B	0.2446	0.6594	0.5860	0.096*	
H7C	0.3880	0.5846	0.5948	0.096*	
C8	0.48007 (15)	0.1666 (3)	0.58408 (11)	0.0699 (5)	
H8A	0.4495	0.0354	0.5873	0.105*	
H8B	0.5676	0.1650	0.5719	0.105*	
H8C	0.4252	0.2349	0.5343	0.105*	
N1	−0.12619 (11)	0.07657 (17)	0.61267 (8)	0.0481 (3)	
N2	0.00157 (12)	0.0812 (2)	0.80881 (8)	0.0569 (3)	
H2	−0.0816	0.0792	0.8041	0.068*	
N3	0.19059 (11)	0.09392 (17)	0.76437 (7)	0.0473 (3)	
O1	−0.17315 (10)	0.07294 (17)	0.52580 (7)	0.0654 (3)	
O2	−0.20198 (10)	0.0741 (2)	0.67215 (8)	0.0725 (4)	
O3	0.26831 (8)	0.39078 (13)	0.63099 (6)	0.0476 (3)	
O4	0.47709 (8)	0.26264 (15)	0.67127 (6)	0.0524 (3)	

### Atomic displacement parameters (Å^2^)

**Table:**

	*U*^11^	*U*^22^	*U*^33^	*U*^12^	*U*^13^	*U*^23^
C1	0.0413 (6)	0.0489 (7)	0.0367 (6)	−0.0019 (5)	0.0030 (5)	0.0045 (5)
C2	0.0448 (6)	0.0371 (6)	0.0384 (6)	−0.0040 (5)	0.0029 (5)	0.0046 (5)
C3	0.0749 (10)	0.0563 (9)	0.0359 (6)	−0.0084 (7)	0.0049 (6)	0.0040 (6)
C4	0.0647 (9)	0.0703 (10)	0.0380 (7)	−0.0080 (7)	−0.0082 (6)	0.0071 (6)
C5	0.0418 (6)	0.0450 (7)	0.0487 (7)	−0.0006 (5)	0.0001 (5)	−0.0001 (5)
C6	0.0349 (6)	0.0491 (7)	0.0379 (6)	−0.0021 (5)	−0.0001 (4)	−0.0028 (5)
C7	0.0774 (11)	0.0510 (9)	0.0627 (9)	−0.0060 (7)	0.0084 (8)	0.0084 (7)
C8	0.0467 (8)	0.1033 (13)	0.0602 (9)	0.0017 (8)	0.0108 (7)	−0.0211 (9)
N1	0.0461 (6)	0.0522 (7)	0.0437 (6)	−0.0003 (5)	0.0008 (5)	0.0042 (5)
N2	0.0519 (6)	0.0807 (9)	0.0379 (6)	−0.0050 (6)	0.0073 (5)	0.0037 (6)
N3	0.0450 (6)	0.0570 (7)	0.0368 (5)	−0.0083 (5)	−0.0013 (4)	0.0060 (5)
O1	0.0562 (6)	0.0879 (8)	0.0445 (5)	0.0038 (5)	−0.0124 (4)	0.0015 (5)
O2	0.0446 (6)	0.1143 (11)	0.0590 (6)	−0.0025 (6)	0.0096 (5)	0.0061 (6)
O3	0.0438 (5)	0.0477 (5)	0.0479 (5)	−0.0010 (4)	−0.0014 (4)	0.0026 (4)
O4	0.0342 (5)	0.0713 (7)	0.0495 (5)	−0.0050 (4)	0.0010 (4)	−0.0086 (5)

### Geometric parameters (Å, °)

**Table:**

C1—N1	1.3447 (17)	C5—H5B	0.9700
C1—C2	1.4131 (17)	C6—O4	1.4028 (15)
C1—H1A	0.9300	C6—O3	1.4036 (15)
C2—N2	1.3282 (17)	C6—H6	0.9800
C2—N3	1.3339 (16)	C7—O3	1.4159 (18)
C3—N2	1.4573 (17)	C7—H7A	0.9600
C3—C4	1.516 (2)	C7—H7B	0.9600
C3—H3A	0.9700	C7—H7C	0.9600
C3—H3B	0.9700	C8—O4	1.4265 (18)
C4—N3	1.4711 (17)	C8—H8A	0.9600
C4—H4A	0.9700	C8—H8B	0.9600
C4—H4B	0.9700	C8—H8C	0.9600
C5—N3	1.4485 (18)	N1—O2	1.2647 (15)
C5—C6	1.5104 (18)	N1—O1	1.2658 (14)
C5—H5A	0.9700	N2—H2	0.8600
			
N1—C1—C2	121.87 (12)	O4—C6—H6	108.2
N1—C1—H1A	119.1	O3—C6—H6	108.2
C2—C1—H1A	119.1	C5—C6—H6	108.2
N2—C2—N3	110.00 (11)	O3—C7—H7A	109.5
N2—C2—C1	126.55 (12)	O3—C7—H7B	109.5
N3—C2—C1	123.45 (12)	H7A—C7—H7B	109.5
N2—C3—C4	102.41 (11)	O3—C7—H7C	109.5
N2—C3—H3A	111.3	H7A—C7—H7C	109.5
C4—C3—H3A	111.3	H7B—C7—H7C	109.5
N2—C3—H3B	111.3	O4—C8—H8A	109.5
C4—C3—H3B	111.3	O4—C8—H8B	109.5
H3A—C3—H3B	109.2	H8A—C8—H8B	109.5
N3—C4—C3	103.81 (11)	O4—C8—H8C	109.5
N3—C4—H4A	111.0	H8A—C8—H8C	109.5
C3—C4—H4A	111.0	H8B—C8—H8C	109.5
N3—C4—H4B	111.0	O2—N1—O1	119.47 (11)
C3—C4—H4B	111.0	O2—N1—C1	121.53 (11)
H4A—C4—H4B	109.0	O1—N1—C1	119.00 (12)
N3—C5—C6	113.17 (11)	C2—N2—C3	112.80 (12)
N3—C5—H5A	108.9	C2—N2—H2	123.6
C6—C5—H5A	108.9	C3—N2—H2	123.6
N3—C5—H5B	108.9	C2—N3—C5	127.22 (11)
C6—C5—H5B	108.9	C2—N3—C4	110.95 (11)
H5A—C5—H5B	107.8	C5—N3—C4	121.47 (11)
O4—C6—O3	112.25 (10)	C6—O3—C7	112.24 (10)
O4—C6—C5	112.21 (11)	C6—O4—C8	115.70 (10)
O3—C6—C5	107.61 (9)		
			
N1—C1—C2—N2	0.7 (2)	C1—C2—N3—C5	−4.8 (2)
N1—C1—C2—N3	−179.68 (12)	N2—C2—N3—C4	1.74 (15)
N2—C3—C4—N3	0.09 (15)	C1—C2—N3—C4	−177.89 (12)
N3—C5—C6—O4	158.56 (10)	C6—C5—N3—C2	105.58 (14)
N3—C5—C6—O3	−77.48 (13)	C6—C5—N3—C4	−81.94 (15)
C2—C1—N1—O2	0.51 (19)	C3—C4—N3—C2	−1.09 (15)
C2—C1—N1—O1	−179.59 (12)	C3—C4—N3—C5	−174.68 (12)
N3—C2—N2—C3	−1.71 (16)	O4—C6—O3—C7	−62.38 (14)
C1—C2—N2—C3	177.90 (12)	C5—C6—O3—C7	173.69 (12)
C4—C3—N2—C2	0.95 (16)	O3—C6—O4—C8	−61.01 (16)
N2—C2—N3—C5	174.88 (12)	C5—C6—O4—C8	60.33 (16)

### Hydrogen-bond geometry (Å, °)

**Table:**

*D*—H···*A*	*D*—H	H···*A*	*D*···*A*	*D*—H···*A*
N2—H2···O2	0.86	2.09	2.6394 (17)	121
N2—H2···O3^i^	0.86	2.64	3.3554 (16)	141

Symmetry codes: (i) −*x*, *y*−1/2, −*z*+3/2.
